# Supplementary material for: Sub-ångström resolution ptychography in a scanning electron microscope at 20 keV
Source: Nat Commun. 2025 Oct 14;16:8977. doi: 10.1038/s41467-025-64133-3 (PMC12521724; doi:10.1038/s41467-025-64133-3)
Supplement: Supplementary file 1 — Supplementary Information [file 41467_2025_64133_MOESM1_ESM.pdf]

SUPPLEMENTARY INFORMATION

Supplementary Figures

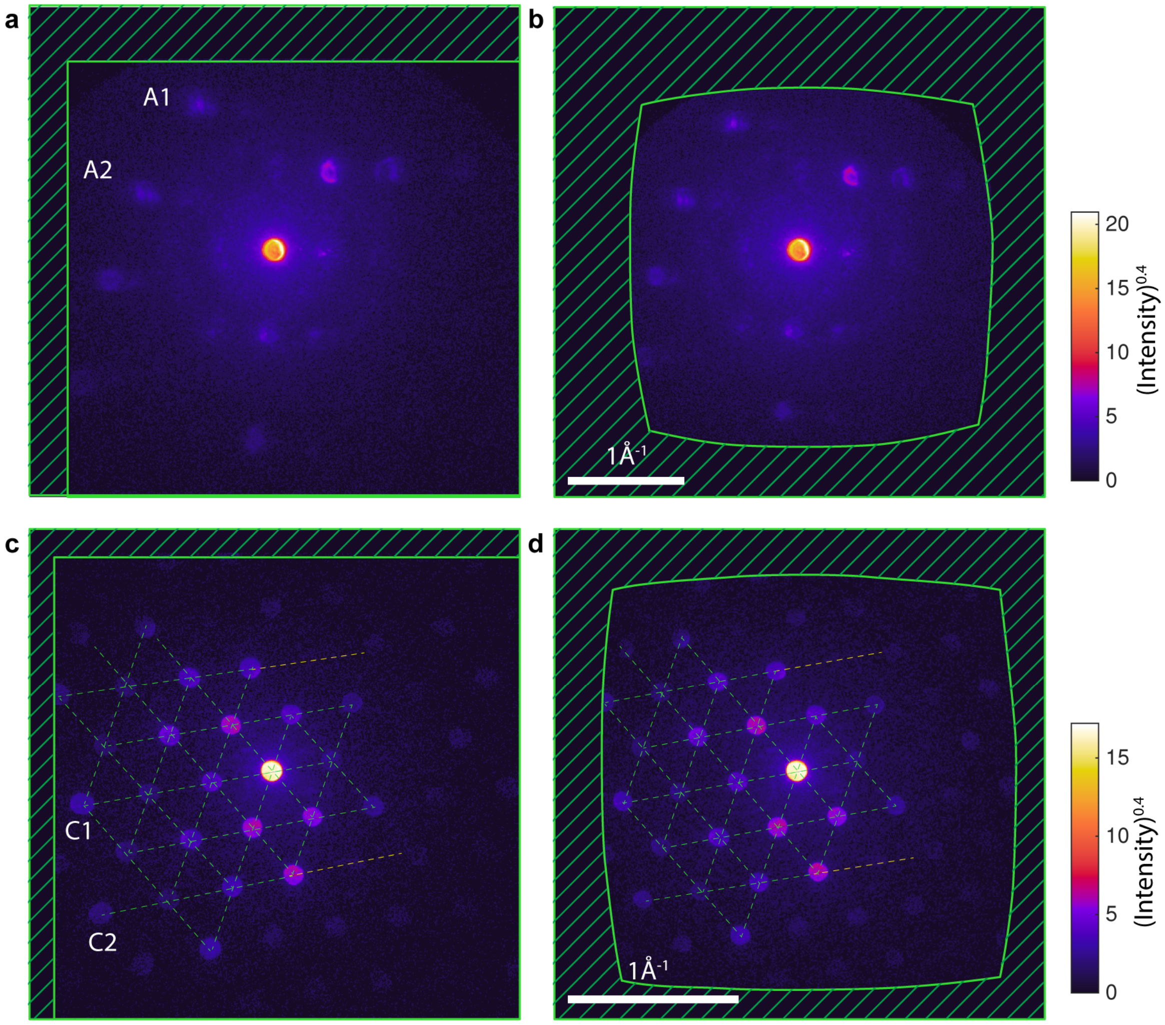

Supplementary Figure 1 – **Sample diffraction data**. **a**, Example of digitally recentered, but otherwise as-collected, electron diffraction data from gold particles on amorphous carbon (Au/aC), and from (**c**) gold islands on few layer MoS_2 (Au/MoS_2). (**b**, **d**) The same data as in (**a**, **c**) after distortion correction. Typical outermost diffraction discs, (A1, A2, C1 and C2) are weak in intensity and do not have sharp edges, uniform inner intensity and are oval-like in shape. Guide to the eye lines (dashed green) are placed over the approximate centres of the stronger diffraction discs in **c** and **d**. Observation that the lines are non-parallel in **c** and parallel in **d** is aided by extending some lines (in dashed yellow). Also, the linear alignment of the discs in **d** indicates that any spiral distortion present is undetectable to the eye. The region hatched in green in each sub-figure does not represent experimental data and is referred to as the mask in the main text. In this region pixels are allowed to float, so there is no penalty in the optimization for model diffraction patterns not being equal to zero with the mask. Scale bars are not given for **a** and **c** as pincushion distortion in these images means a linear scale cannot be used. However, in **a** and **c** the distance to the innermost, lowest order, diffraction discs were taken to be the same as in the non-distorted case for the purpose of measuring, *s*, the radial coordinate in the distorted images.

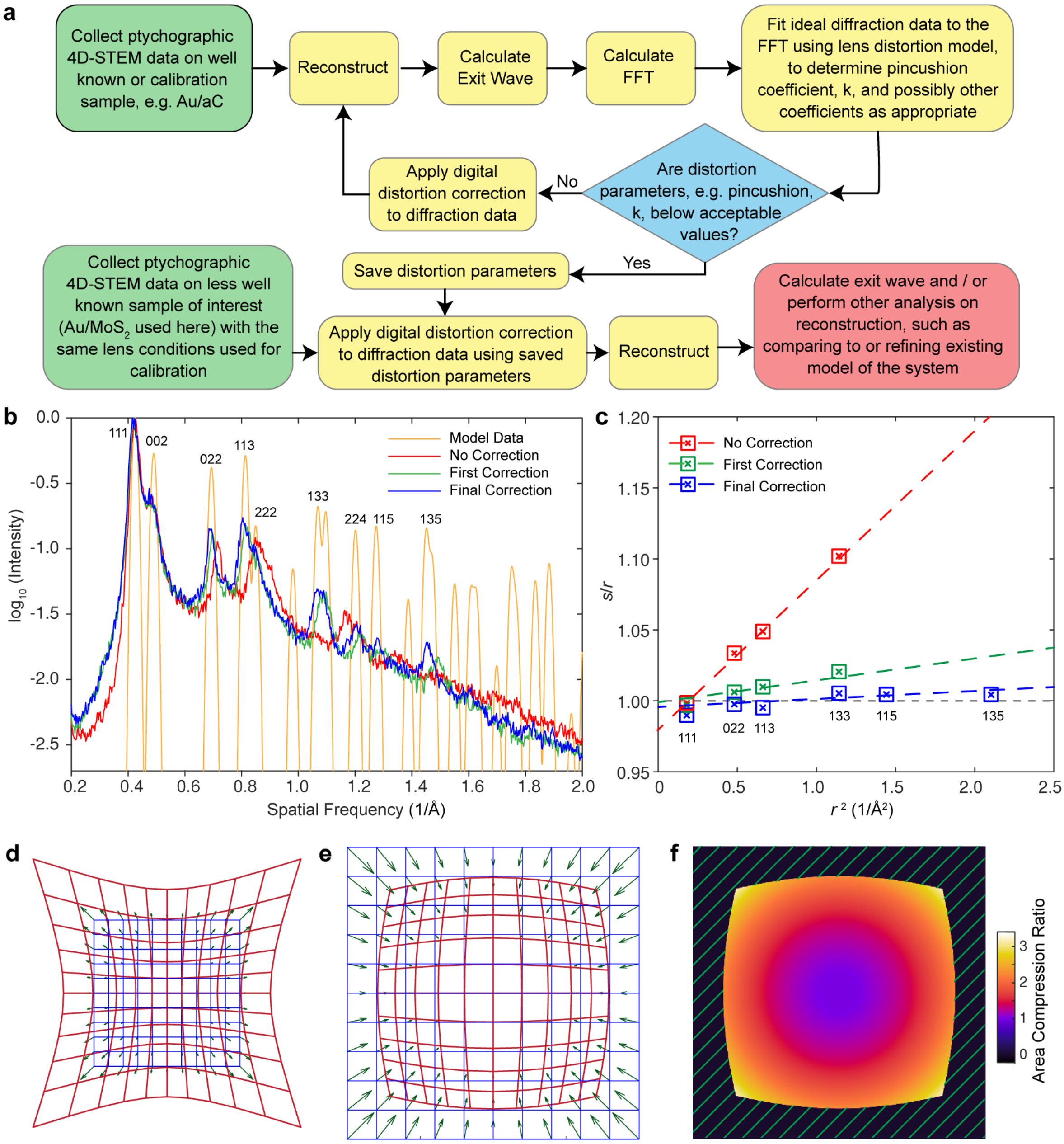

Supplementary Figure 2 – **The process and results of distortion correction**. **a**, Flow diagram of the reconstruction process incorporating lens distortion determination and digital correction. **b**, Plots of the average radial FT profile of the reconstructions produced from (red line) the as-collected diffraction data; (green line) the initially corrected diffraction data; (blue line) the final corrected diffraction data; and (orange) model diffraction data generated for a sample comprised of gold particles. The dominant peaks are labelled with their related diffraction plane families. **c**, Plots showing the relationship between r the expected non-distorted spatial frequency, and s the as measured spatial frequency of the dominant diffraction peaks measured from the radial average of the FT of reconstruction, where the measurement for s was taken by taking the {111} peak spatial frequency as fixed. The linear fits (dashed lines) indicate the distortion is primarily pincushion having the form of Equation 1 in the Methods. **d**, Ideal diffraction data, mapped over the regular square blue mesh, is distorted through our lens system to the red mesh which has a pincushion like form. **e**, Distorted as-collected data mapped over the blue grid needs to be mapped to the red grid to be distortion free. In **e** note that, for example, the uppermost right-hand grid square is larger than the uppermost right-hand red square it is mapped to. To preserve total counts in the diffraction data, the intensity is adjusted in line with the area ratio given in Equation S.5 in the Supplemental Information. **f**, The resulting area compression ratio, which would arise from applying the transform in **e** to a uniform unit value image. The region hatched in green, coming from outside of the original image, is set to zero. If the image in **f** is N-by-N pixels, the sum of all pixel values **f** will thus be N^2.

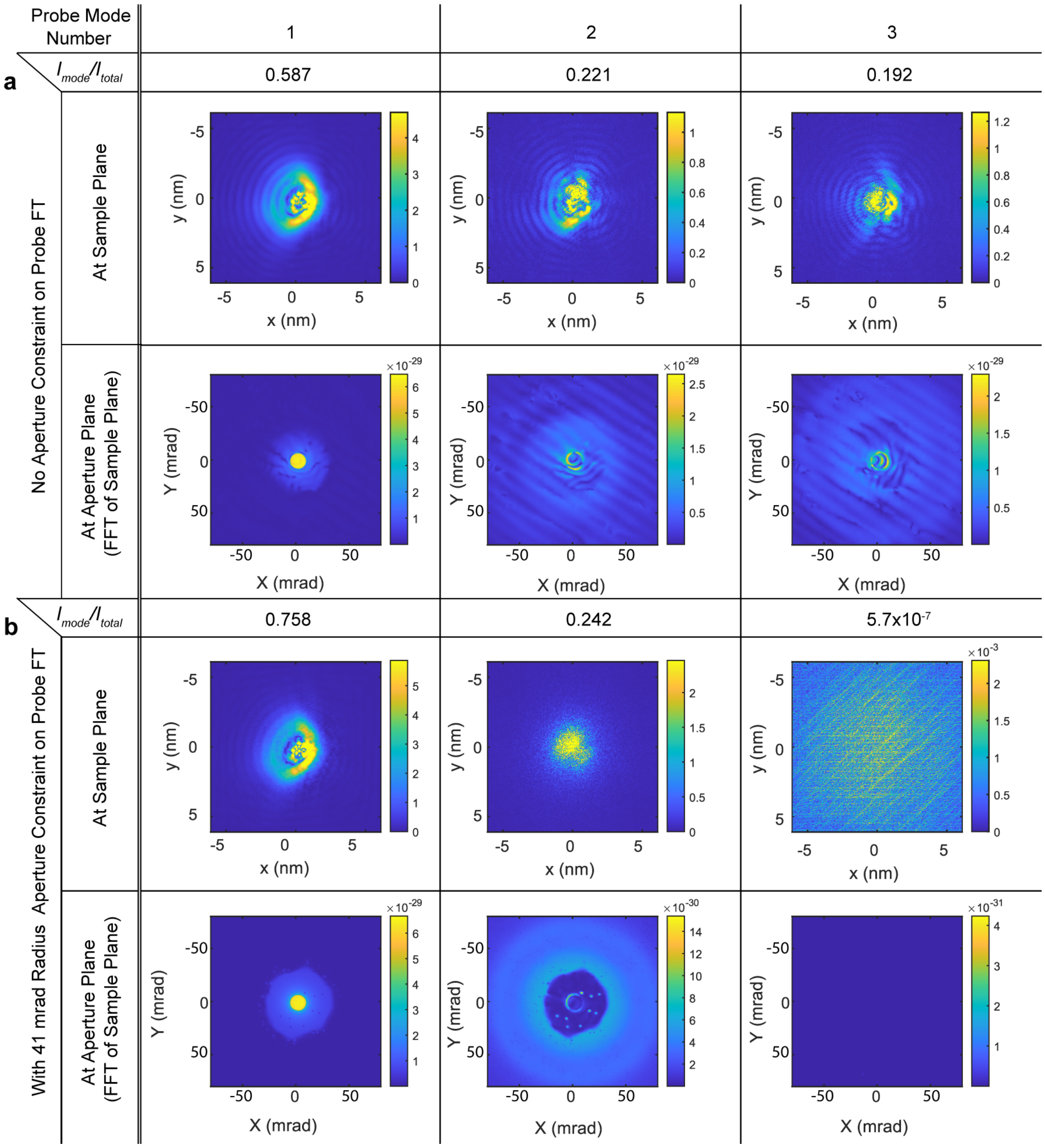

Supplementary Figure 3 – **Probe models produced from the Au/aC reconstruction**. Here 3 probe modes were used and the fraction of the intensity in each mode is given at the top of each column in the table as the ratio I_{mode}/I_{total}.for reconstructions with (**a**) no aperture constraint and (**b**) with an aperture constraint. Each column presents a mode, and the upper row of each table sub-part (**a**, **b**) presents the amplitude of the complex wavefunction representing the probe at the sample; and the lower row shows the amplitude at the aperture plane, found by taking an FT of the probe.

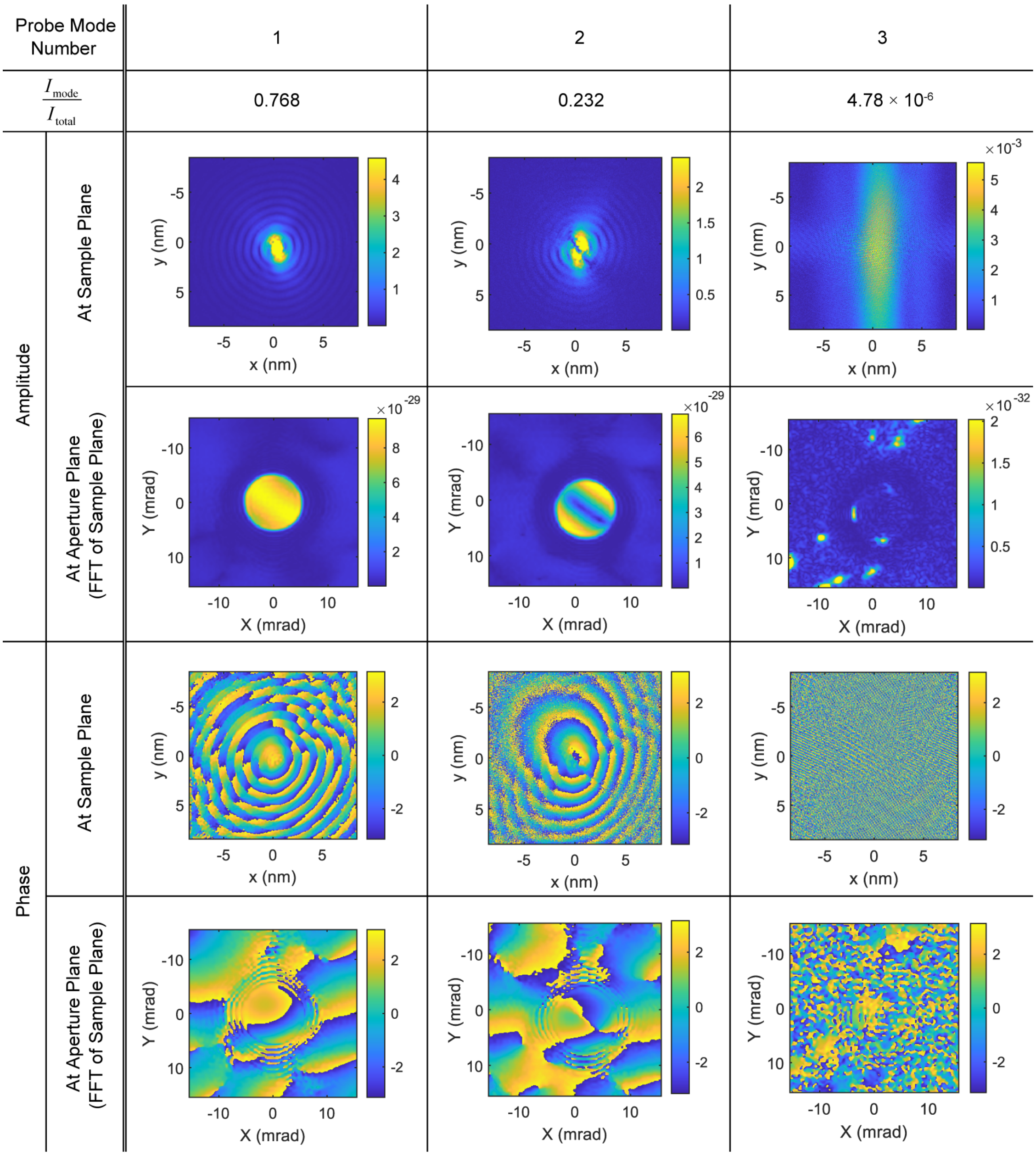

Supplementary Figure 4 – **Probe model produced from the Au/MoS_2 reconstruction**. Here 3 probe modes were used and the fraction of the intensity in each mode is given at the top of each column as the ratio I_{mode}/I_{total}. Each column presents a mode, and the rows present: (rows 1 and 3, respectively) the amplitude and phase of the complex wavefunction representing the probe at the sample; and (rows 2 and 4, respectively) the amplitude and phase of the probe at the aperture plane, found by taking an FT of the complex probe presented in rows 1 and 3.

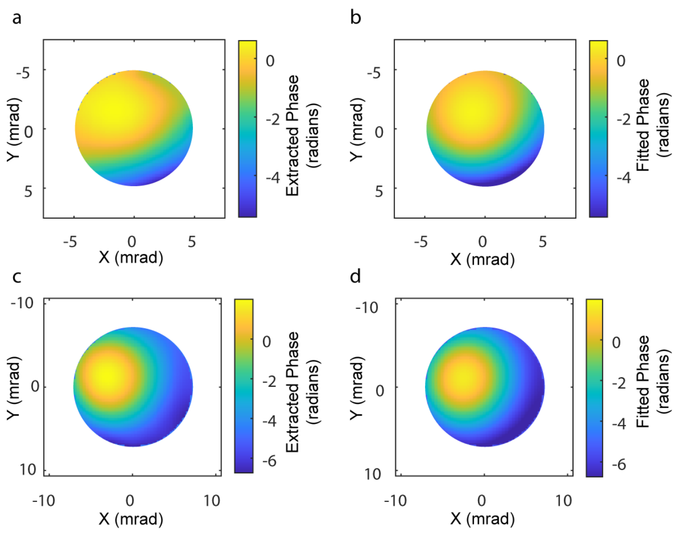

e

Extracted (Fitted) Aberration or Parameter	Au/aC	Au/MoS_2
Piston (pm)	7.8	7.3
T_x (nm)	-1.20	-0.37
T_y (nm)	-0.52	-0.57
Defocus (nm)	-392	-386
A_{1x} (nm)	26	40
A_{1y} (nm)	15	-25
B_{2x} (µm)	16.6	4.4
B_{2y} (µm)	5.3	-8.3
C_s (mm)	3.9	3.8
Aperture Cutoff Angle (mrad)	7.2	5.0

Supplementary Figure 5 – **Unwrapped phase of reconstructed probes at the aperture plane and fitted phase**. (**a**, **c**) The unwrapped inverted phase for the probe at the aperture plane extracted from the (**a**) Au/aC and (**c**) Au/MoS_2 reconstruction and (**b**, **d**) their respective fitted phases. **e**, Table showing the aberration coefficients used to produce the fitted models. The coefficients are described in the Supplemental Information, Equation S.6.

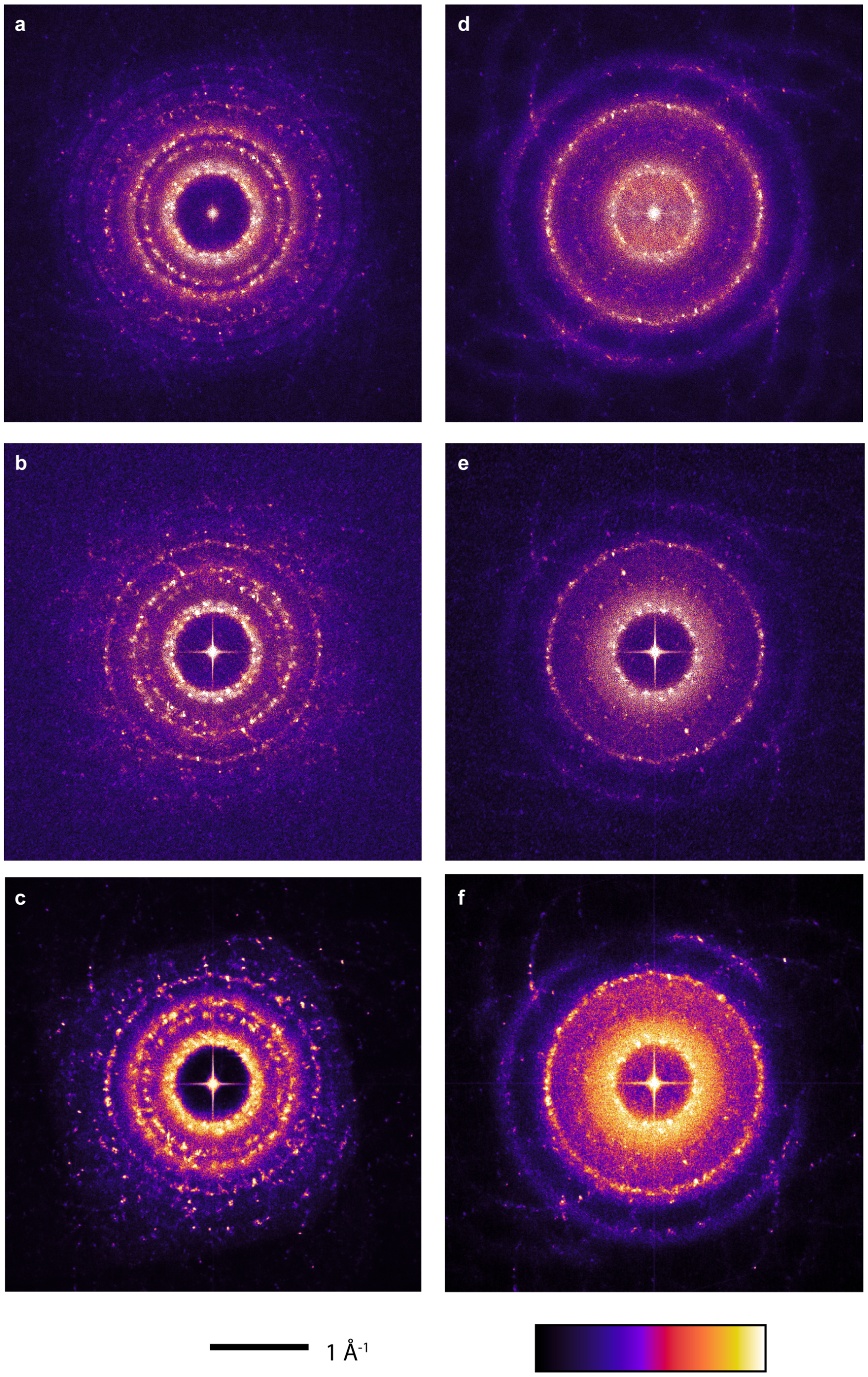

Supplementary Figure 6 – **Presentations of the Fourier Transforms (FTs) from processing in a variety of ways the multi-slice reconstruction of Au/aC**. The amplitude of the FT of the: **a**, exit-wave intensity (square of the amplitude); **b**, exit-wave phase, where the phase is represented as a real number before taking its FT; **c**, complex exit-wave; **d**, square of the amplitude of the product of the amplitude of the individual slices; **e**, sum of the phases of the individual slices; **f**, complex product of the individual complex slices. Note that (**a** – **c**) give ring intensities that correctly indicate randomly oriented Au particles, whereas presentations (**d** – **f**) would naively be taken to incorrectly indicate a preferred orientation or structural anomaly for the particles (see also Supplementary Information). In all images the gamma is adjusted to 0.9, upper and lower 1% of pixel values are saturated. Scale bar is 1 Å^{-1}, color scale is as depicted at base of figures. In parts **a** and **d** a Hann window was applied to the image intensities prior to taking the FT.

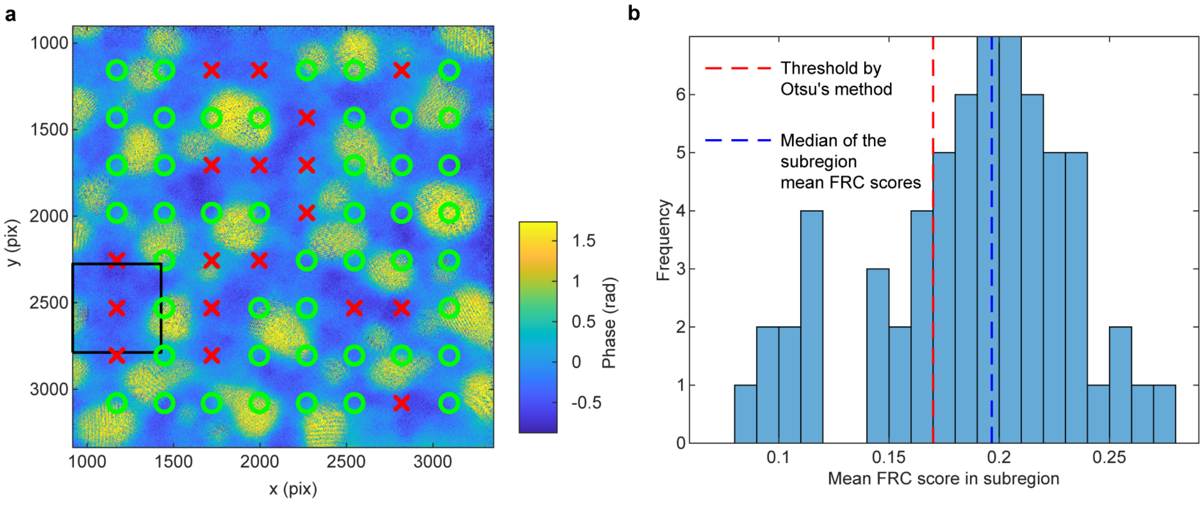

Supplementary Figure 7 – **Sub-regions used in the mean FRC characteristic**. **a**, The reconstructed phase is overlaid with markers showing the centres (green circles) of sub-regions included in the mean FRC characteristic given in Fig. 2. Excluded regions are shown with a red cross. The size of the subregions is 512-by-512 pixels. **b**, Histogram of the mean FRC scores in each subregion. The threshold given by Otsu's method is marked with the red dashed line. Regions with a mean FRC above this threshold (green circles in **a**) are included in average FRC (Fig. 2). Regions below the threshold are dominated by amorphous carbon, such as in the example excluded subregion indicated by the black square in **a**.

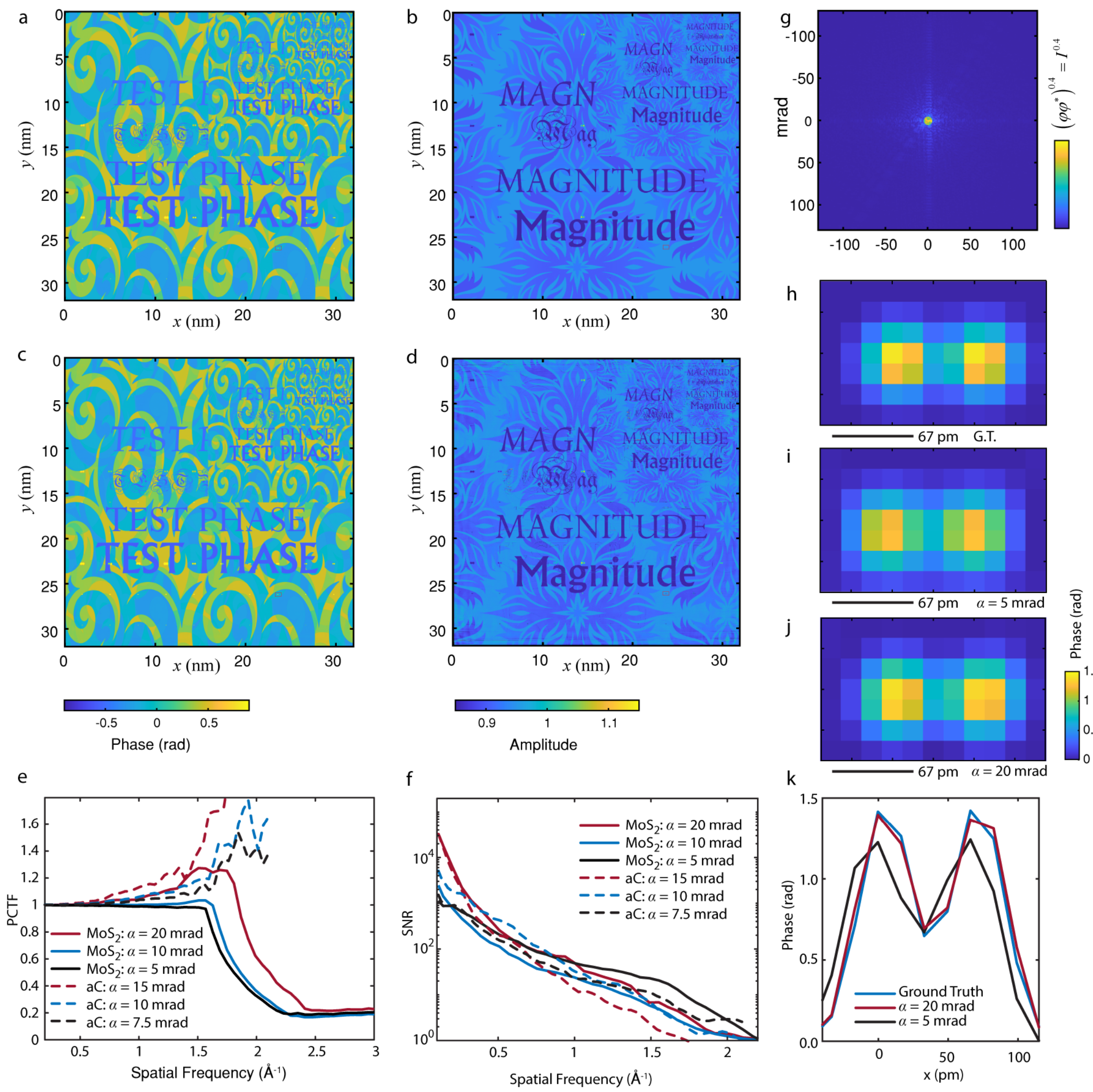

Supplementary Figure 8 – Characteristics of reconstructions performed on a synthetic single-slice object using similar reconstruction parameters as the experimental data reconstructions. **a** The single-slice synthetic test object phase and **b**, the test object amplitude. A reconstruction (**c**, **d** showing phase and amplitude respectively) created from simulated diffraction using the same beam energy (20 keV), beam defocus, reconstructed pixel size, beam convergence angle (5 mrad), probe overlap, and recipe parameters as those used for Au-MoS_2 reconstruction (see Supplementary Information and Supplemental Data Table 1), though with adjustments for a single slice model. Comparisons between the ground truth test object and reconstructions for a range of beam convergence half-angles (α) and for the Au-aC recipe yield **e** the phase contrast transfer function (PCTF) and **f** the signal to noise ratio (SNR). The solid and dashed lines in **e**, **f** represent the reconstruction recipes used for the Au-MoS_2 and Au-aC datasets respectively, with the black data-lines giving characteristics for the α used in the experiments. Our FRC determined resolution corresponds to approximately 1.5 $Å^{-1}$. **g**: An example simulated diffraction pattern, used in creating the reconstruction shown in **c**, **d**. The test object (**a**, **b**) contains isolated double gaussian peak features impressed upon the phase, the amplitude, or a combination of both. An example phase-only pair of peaks, taken from coordinates (x = 24 nm, y = 26 nm in a and therein highlighted with a red rectangle) is shown in **h**. The phase of the reconstructions of the region shown **h**, using α = 5 mrad and 20 mrad for the Au-MoS_2 reconstruction recipe are shown in **i** and **j** respectively. **k**: Profiles of the phase taken in x-direction through the centres of the peaks shown in **h**, **i** and **j**.

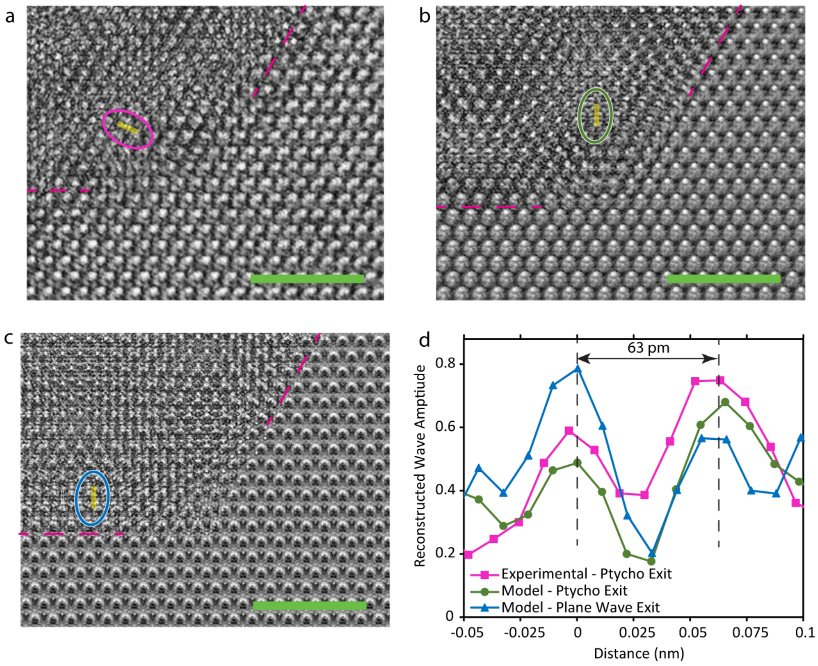

Supplementary Figure 9 - **Amplitude of exit wave reconstructions for the experimental and simulated Au-MoS_2 datasets**. **a, b:** Amplitude of the exit wave from the ptychographic reconstructions of (**a**) the experimental Au-MoS_2 data and (**b)** from a multi-slice model of a tapered-edge Au island on MoS_2. **c:** The exit wave produced from assuming parallel illumination on the multi-slice sample model that was used to produce the diffraction dataset for the reconstruction shown in **b**. In the images (a, b, c) the gold island is in the upper left has a tapered edge, which terminates approximately at the magenta dashed line. The scale bar (lower right, green) in each image is 2 nm. **d**: Profiles of the amplitude taken through regions of the reconstructions or images (highlighted yellow, and surrounded with an ellipse in **a**, **b**, and **c**). The image and profiles show some lattice like fringes separated by approximately 63 pm, corresponding to some of the highest spatial frequencies present in Figure 3b.

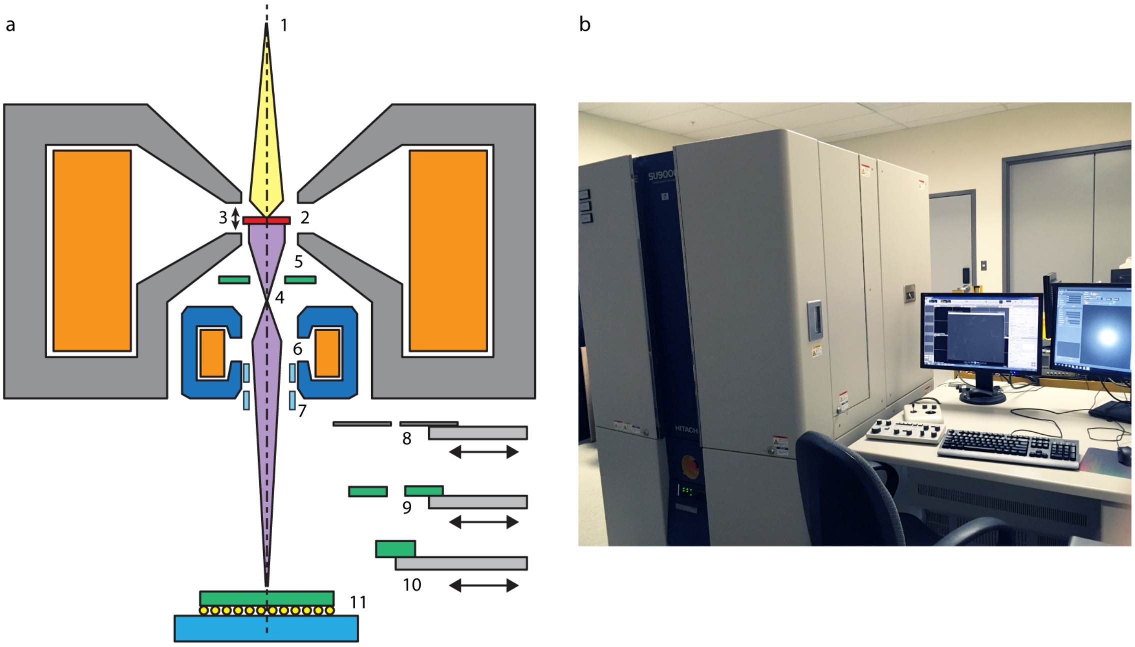

Supplementary Figure 10 – **Arrangement of the objective – projector lenses in the experimental scanning electron microscope. a:** The schematic cross-section shows the electron beam, with (1) an upstream cross-over, being focussed by (2) the magnetic field of the objective lens on to (3, shown colored red) the sample which can be adjusted in height. Altering the objective lens strength and the sample height adjusts the position of (4) a down-stream beam cross-over and the angular detection range for (5) a fixed position high angle annular dark field (HAADF) detector. The beam emerging from the sample (shaded purple), then passes through (6) a single magnetic projector lens, and (7) a set of beam deflection coils. Below the lens and coils is an insertable (8) bright field aperture, (9) low angle annular dark field (LADF) detector, and (10) bright-field detector. With the above detectors (8-10) retracted, electrons then impact directly on (11) the pixelated direct detector (Dectris Quadro) used in this work. **b**: A photo of the experimental instrument. The sample is loaded at the desktop level and so most of the cross-section shown (**a**) sits below the desktop level, within the column cabinet to the left of the photograph.

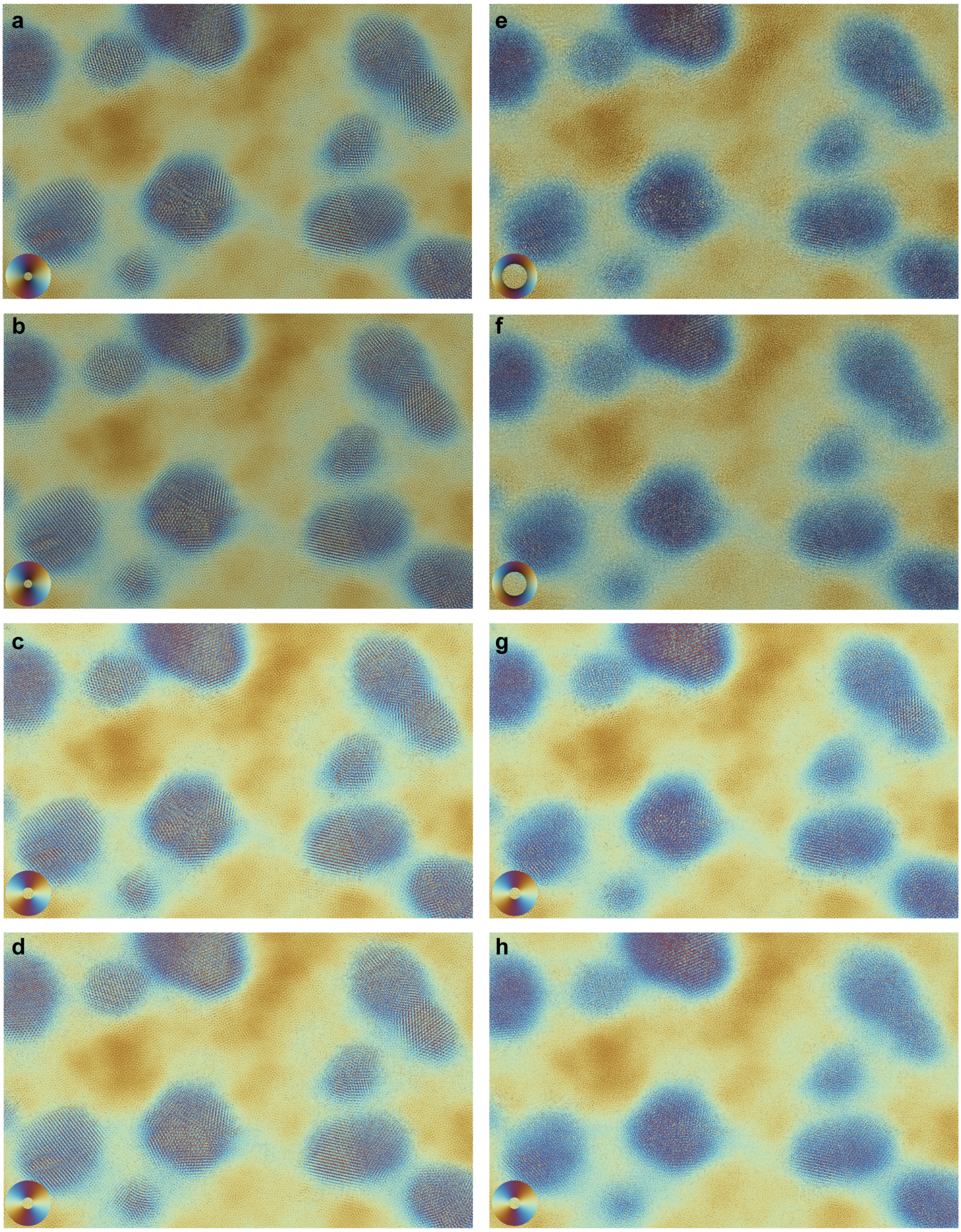

Supplementary Figure 11 – **Reconstructions of Au particles on amorphous carbon (a, c, e, g) without and (b, d, f, h) with an aperture constraint on the FT of the probe model**. Presentations are made of (**a** – **d**) the exit wave found by numerically propagating an electron plane wave through the object model slices and (**e** – **h**) taking the sum of the unwrapped phases and product of the amplitudes. Parts **a**, **b**, **e** and **f** show the result using a mixed phase – amplitude color-scale, whereas **c**, **d**, **g** and **h** show the phase only. The color scales are given in the lower left of each figure part, where one full cycle around the color wheel's azimuthal angle represents the phase, spanning 0 – 2π. Where amplitude is presented, the radial coordinate of the color-wheel spans are **a**:[0.43, 2.34], **b**:[0.43, 2.35], **e**:[0.98, 1.79] and **f**:[0.95, 1.80]. The width of the field of view in each sub-figure is 38 nm. Part **b** is an enlarged region of Fig. 1a.

Notes (Row #)	Sample Material	Gold particles on amorphous carbon (Au/aC)				Gold islands on MoS_2 (Au/MoS_2)				
1	Recipe Step	1	2	3	4	1	2	3	4	5
2	Algorithm	ePIE	MLs	MLs	MLs	ePIE	MLs	MLs	MLs	MLs
3	Number of Iterations	50	300	300	300	50	300	300	400	1000
4	Grouping	1	40	40	40	1	40	40	40	20
5	Diffraction Size (pixels, $x \times x$, □)	512	512	512	512	512	512	512	512	1024
6	Probe Modes	1	3	3	3	1	1	3	3	3
7	Probe Update	0.1	0.005	0.005	0.005	0.05	0.005	0.005	0.005	0.005
8	Background Fitting	No	No	No	No	No	Yes	Yes	Yes	Yes
9	Background Width	-	-	-	-	-	3	3	3	3
10	Initial Probe Mask Normalized Radius	-	0.9	-	-	-	0.9	-	-	-
11	Aperture Constraint Normalized Radius	-	0.9	0.46	0.23	-	0.4	0.2	0.1	0.1
12	Layers	1	1	2	4	1	1	2	4	4
13	Layer Thickness (Å)	-	-	20	20	20	20	20	20	20
14	β	0	0.5	0.5	0.5	0	0.5	0.5	0.5	0.5
15	p	0	0.1	0.1	0.1	0	0.1	0.1	0.1	0.1
16	Regularization	0	0.005	0.005	0.005	0	0.05	0.05	0.05	0.05
17	m	0	0	0	0	0	0	0	0.03	0.03
18	Reconstructed Pixel Size (pm)	23.8				33.14				16.57
19	Probe Guess: Defocus (nm)	-370				-400				
20	Probe Guess: C_s (mm)	3.2				3.2				
21	Final Fourier Error (Result - not recipe parameter]	2.406	0.953	0.935	0.912	1.830	0.860	0.879	0.838	0.782*

Supplementary Table 1 – Reconstruction Recipe Parameters. See Supplemental Information, Section 1 for notes and details.

1. Reconstruction

1.1 Recipes and Parameters

Our reconstruction process used multiple algorithms and recipes, where the probe and object models resulting from a recipe were passed to the next recipe, indicated by the successive recipe step numbers in row 1 of Supplementary Table 1. In transitioning recipes only the probe and object were transferred and other intermediates or terms involving gradients of updates, momentum etc., were reset. We used two main algorithms: the extended ptychographical iterative engine (ePIE)[26]; the iterative least-squares solver for generalized maximum-likelihood ptychography (LSQ-ML)[63], and a multi-slice adaptation of (MS-)LSQ-ML, which was implemented within a modified version of PtychoShelves[19]. When using the LSQ-ML algorithms we used a sparse grouping method and referred to the method as MLs in row 2, with the trailing 's' indicating sparse grouping. The number of diffraction patterns within a sparse group is given in row 4 of Supplementary Table 1. In the latter recipe steps three incoherent probe modes[53] were introduced (row 6). In all cases our object update factor was kept at 1, though the probe update factor was varied as listed in Table 1 row 7. However, in the case of the MLs solver, note that the probe update factor is modified to an optimal value as described by Odstrčil *et al*[63] using the β factor given in row 14, as implemented within Ptychoshelves[64] as the '`beta_lsq`' parameter. Background fitting and estimation as implemented in PtychoShelves[64] was used in the latter stages of the Au/MoS_2 reconstruction as in row 8. Here, the background model was considered to have a form proportional to a 'blurred' version of the mean diffraction pattern, where the blurring convolution function had the from

$$\exp\left(-\sqrt{\frac{x^2+y^2}{w^2}}\right)=\exp\left(-\frac{r}{w}\right). \tag{S.1}$$

Here x and y are the column and row pixel distances respectively from the centre of the convolution kernel, r is the radial distance from the centre, and w is the width parameter (in pixel units) given in row 9. A scaled version of this background is added or subtracted as appropriate to the expected diffraction patterns produced from considering the incoherent sum of the diffraction produced from the probe illumination modes, to better match the experimental intensity.

When proceeding from step 1 to 2 with both specimens, convergence and error metrics were improved by masking the probe model produced from ePIE with a circular mask before transferring to the subsequent MLs step. The mask did not alter the probe model within a radius of 0.9 × (512/2) pixels from the centre, but set the pixels outside this radius to a value of zero. The mask on the probe (applied in real space, not the detector plane), was applied in the first iteration, and is given in row 10 as 'Initial Probe Mask Normalized Radius'. In subsequent steps, a constraint was placed on the FT of the probe (at the detector or aperture plane) that forced

the pixels outside a certain normalized radius towards zero on each iteration. This reflected the fact that we knew our probe was physically limited by a hard (circular) aperture at this plane. As an example, in step 4 of the MoS_2 reconstruction, a normalized radius of 0.1 (shown in row 11) represents a mask of radius 0.1 λ / (2 × reconstructed pixel size) = 13 mrad, whereas in step 5 this becomes approximately 26 mrad. As our beam angle was expected and shown through the reconstruction to have a beam half-angle of 5 mrad, these limits of 13 and 26 mrad did not unduly constrain the reconstruction, though helped direct it towards a physical solution.

As reported, our solution used multiple slices. We progressively increased the number of slices in successive recipe steps as shown in row 12. In each subsequent recipe step of the slice doubling process, the phase of each slice from the prior step was unwrapped and then equally divided between the nearest two slices in the new step. Similarly, the square root of the amplitude was taken and given to the nearest two slices when going from one step to another, when the number of slices doubled.

Row 15 gives p as the damping factor for the MLs method, related to preconditioning its update, as defined as the parameter `par.delta_p` in Ptychoshelves.

Row 16 gives the regularization factor, R, as defined in Ptychoshelves in the parameter `eng.regularize_layers`, where $0 < R << 1$. If $R = 0$, no regularization is performed, whereas if $R = 0.01$ weak regularization is performed that will slowly symmetrize information content between layers. Relatively weak regularization (R = 0.005) was applied for Au/aC, and stronger regularization (R = 0.05) was applied for Au/MoS_2, such that the algorithm converged to a stable solution.

Row 17 gives the parameter that controls an additional simple weak regularization method that smooths only the amplitude of the complex object by convolving it with a 3-by-3 voxel kernel, where the centre pixel has the value μ (given in the table), and the all the other kernel voxels are 1.

Row 18 gives the reconstructed pixel size in pm. The reconstructed pixel size d is

$$d = \frac{\lambda}{\Omega}, \tag{S.2}$$

where λ is the electron wavelength, and Ω is the angular field of view on pixelated detector at the diffraction plane[15]. Therefore we re-evaluated our reconstructed pixel size after applying distortion correction. The tabulated values represent the final values after corrections to the diffraction pattern were applied.

Rows 19 and 20, give the defocus and spherical aberration coefficient used to create our initial probe guess. These were initially estimated from our knowledge of the SEM and applied defocus of the objective lens. After initial reconstructions, we extracted probe aberration coefficients from the reconstructed probes (see next section). The extracted aberration coefficients were then used to guide our initial probe guess. However, we did not use the exact aberration values extracted from the final reconstruction step to create the initial probe guess

to maintain a realistic situation where the probe is to some degree unknown before commencing the reconstruction.

We show in Row 21 the resulting median Fourier Error (FE) for each reconstruction step which is a result of the reconstruction step and is not a step setup parameter. The Fourier error for a particular diffraction pattern is defined as the mean Euclidean distance between the experimental measured (Ψ_i) and model reconstructed diffraction data pixels (φ_i) as

$$FE = \sqrt{\frac{\sum_i \left(\Psi_i - \varphi_i\right)^2}{N}}. \tag{S.3}$$

Pixels are only compared in the region where there are real measurements. Thus, our masked and extrapolated region outside of where our distortion corrected data is present is excluded from comparison: we set $\Psi_i - \varphi_i = 0$ in this region. However, N is kept as the total number of pixels including these masked pixels. For step 5 of the Au/MoS_2 reconstruction we multiplied the Fourier error given by the equation (S.3) above by two (marked* in Row 21) to allow easier comparison to the prior steps. As each individual diffraction patterns has its own FE, we report the median over the entire set in row 21.

Prior works shows that a ptychographic reconstruction is only valid for a coherently scattering object thickness of T, given by the theoretical approximation,

$$T \leq k\left(\delta r\right)^2 / \lambda, \tag{S.4}$$

where δr is the image resolution, λ is the electron wavelength, and k is a constant in the range [2, 5.2][51]. Here, the lower limit of 2 is arrived at through analysis of the Ewald sphere[69], and the upper limit of 5.2 arises through numerical modelling and experimental results (see supplemental material of [70]). With δr = 0.07 nm (our approximate resolution), and a beam energy of 20 keV giving λ = 8.59 pm, we thus should have $T \leq 1.1 - 3.0$ nm for a single slice reconstruction approximation to hold. When the sample has a thickness greater than T, multi-slice ptychographic methods must be used (perhaps in combination tomography[71]). Here, we opted to use $n = 4$ slices (where n is the number of slices), with their separation (T_s) approximately in the middle of range of T, thus setting $T_s = 2$ nm, to give a total acceptable object thickness of $nT_s = 8$ nm.

Matching to our experimental diffraction patterns (see Supplementary Information) indicates that the MoS_2 specimen is composed of 5 layers (3.1 nm), which fits within the thickness limit given by nT_s. For the gold islands, we estimate their thickness by taking their mean phase shift relative to the MoS_2 support from the experimental reconstructions and compare this to the phase shift seen in a reconstruction produced from simulated diffraction patterns for which the thickness of gold is known. Looking at an example island in the top

right of Fig. 3a, we see a mean phase shift relative to the support of approximately 1.6 radians. Our model gold island (Fig. 4) had a peak thickness of 3.4 nm and showed a mean phase shift in its reconstruction (Fig. 4e) of approx. 1.8 radians. Thus, we estimate our experimental gold island to have a thickness of 3.8 nm. This gives a total thickness of 6.9 nm for this region of the Au/MoS_2 reconstruction. Looking at an example Au particle from the Au/aC reconstruction in Fig. 1b, the peak phase shift difference between the particle and the amorphous carbon support is approximately 3 radians. A 20 keV electron beam has an interaction constant of 1.86×10^7 rad V^{-1} m^{-1}, and gold has an estimated mean inner potential of 21.4 eV[72], thus implying a peak gold particle thickness of ~7.5 nm, which is similar to the particle's lateral dimensions, as would be expected for a near spherical particles.

Any hydrocarbon-based contamination on the sample is likely to be amorphous and mobile in nature and thus effectively contribute an incoherent background to the otherwise coherent diffraction data. Incoherent contributions to diffraction data cannot be effectively reconstructed by the ptychographic algorithms. Thus, the thickness limit consideration encapsulated in Equation (S.4) relates only to contiguous regions of the sample or object that scatter coherently: namely, the crystalline or poly-crystalline gold and MoS_2 in our samples.

Consequently, even though our samples likely had an amorphous carbon-based surface contamination layer (and in the case of the Au/aC sample a ~15 nm thickness amorphous carbon support film) that make the total sample thickness values greater than the 6.9 nm and 7.5 nm just determined, the reconstructions retain validity as the coherently scattering thickness regions are less than nT_s (= 8 nm). However, the predominantly incoherent component to our diffraction data produced by the amorphous support or contamination material, likely contributes to intensity observed in the higher order probe model modes (Supplementary Figs. 3 and 4, mode number > 1). This is more so in the absence of incoherent background estimation and removal, which was applied to the MoS_2 reconstruction but not to the Au/aC reconstruction (Supplementary Table 1).

Effects and artefacts resulting from noise-induced random beam-position shifts upon individual reconstructions can be minimized or eliminated using correction algorithms[73,74]. However, it is preferable to minimize or eliminate physical noise sources rather than computationally correct for them to reduce or remove the computational cost and residual uncertainty inherent to the correction algorithms. Thus, here we placed our SEM in a low-noise environment and observed stable, low-noise, high-resolution focussed probe conventional SE imaging prior to performing our defocused-probe data acquisitions. Applying positional correction algorithms[73,74] within our reconstruction process resulted in insignificant changes of our Fourier error metric and did not improve our resolution measure. Thus, we did not apply such correction algorithms in the presented

and analyzed reconstructions, and took the insignificant change of our Fourier error metric as evidence of low scan noise and good beam stability in our SEM.

1.2 Distortion Correction Intensity Correction

Continuing from our methods description in the main text, imagine collecting pincushion distorted diffraction data where every pixel recorded one electron. Correcting the data without intensity adjustment would produce a smaller region of pixels with a value of one, surrounded by a region – such as that shown hatched green in Supplementary Fig. 1(b, d) - with an undefined value (or an arbitrarily assigned value, such as zero). Naively summing the pixel values in the 'corrected' image would then incorrectly indicate a smaller number of electron counts. Accordingly, prior to performing a conventional (non-intensity adjusting) distortion correction mapping we multiply the intensity of source data by

$$\frac{dA_s}{dA_r} = \frac{s d\theta ds}{r d\theta dr} = \frac{s}{r}\frac{ds}{dr} = M^2\left(1+kr^2\right)\left(1+3kr^2\right). \qquad \text{(S.5)}$$

In the manner consistent with that applied to fMRI image correction[66], the correction is determined by considering the ratio of infinitesimal areas of distorted and undistorted image space patches, dA_s and dA_r respectively. The resulting image intensity multiplier, for a typical pincushion distortion coefficient example is presented in Supplementary Fig. 2f. This multiplier is implemented within `lensdistort.m` in the `Runner` directory of the related code repository (https://github.com/ArthurBlackburn/PtychoRunner)[68], with a usage example given in `TestScripts\Part_3_DistortionFit_and_Correct.m`.

2. Probe Reconstructions

Ptychographic reconstructions yield a model of the electron probe. The final probe model's amplitude and phase is visualized at entry to the sample model in the top (amplitude) and third (phase) rows of Supplementary Fig. 3 and Supplementary Fig. 4, for the Au/aC and Au/MoS_2 reconstructions respectively. As noted, our solutions used three probe modes and the relative contribution of each mode to the overall intensity is given in the title of each column as I_{mode}/I_{total}, where I_{mode} is the summed intensity in the mode, I_{total} is summed intensity in all the modes, and intensity is equal to the square of the wave amplitude. The FT of the probe at the sample plane is given in the second and fourth rows of the Figures, showing the amplitude and phase respectively. In both figures, if we look at the first probe mode intensity at the aperture plane (column 1, row 2) we see that as expected most of the intensity is concentrated within a circular region, as expected with the electron beam being

formed with a circular aperture. Some intensity is also present outside the aperture, which most likely represents the effects of scattering from amorphous material and other incoherent effects.

The phase of the probe model at the aperture (or equivalently the diffraction plane) can also be used to approximately determine the aberration coefficients the probe forming system. The unwrapped phase of the dominant probe mode for each model is presented in Supplementary Fig. 4 (a, c) for the Au/aC and Au/MoS_2 reconstructions respectively. We then fit this phase with the first 36 Zernike polynomials, using a Moore-Penrose pseudo-inverse approach (least squares) to determine the coefficients of each polynomial. These coefficients are then used to determine the Seidel aberration coefficients, using relationships given by Tyson[75]. These are then further adjusted, if necessary, with pre-factors to allow the phase-shift at the aperture to be defined using the customary aberration coefficients described by

$$\phi = \frac{2\pi}{\lambda}\begin{pmatrix} P + (T_x\cos(\theta) + T_y\sin(\theta))\alpha + \frac{1}{2}\left(d + A_{1x}\cos(2\theta) + A_{1y}\sin(2\theta)\right)\alpha^2 + \\ \left(B_{2x}\cos(\theta) + B_{2y}\sin(\theta)\right)\alpha^3 + \frac{1}{4}C_s\alpha^4 \end{pmatrix} \quad \text{(S.6)}$$

where ϕ is the phase across the aperture, λ is the electron wavelength, α is the angle from the centre of the aperture, P is the piston, (T_x, T_y) are beam tilt coefficients, d is the defocus, (A_{1x}, A_{1y}) are the x, y components of the primary astigmatism, (B_{2x}, B_{2y}) are x, y components of the coma aberration, and C_s is the spherical aberration. P, of course, does not have any physical consequences, but is included in the definition for completeness. Note that an electron beam of 20 keV energy has wavelength $\lambda = 8.59$ pm. Extracted aberration coefficients are given in the table of Supplementary Fig. 4(e), and the phase shifts which they describe are presented in Supplementary Fig. 4(b, d) for the Au/aC and Au/MoS_2 reconstructions respectively.

3. Propagated Exit Wave and Summed Phase Comparisons

As noted in the main text, producing a simulated exit wave from a multiple slice model produces images and FTs that are more consistent with expectations from conventional TEM imaging. To simulate an exit wave, we assume planar incident illumination on the uppermost slice. At the first slice we multiply this plane illumination with the complex value of the first slice and propagate it to the adjacent slice. At the adjacent slice another multiplication is performed, and the wave is propagated again to the next slice. This is repeated until the last slice, where we finally extract and present the product of wave propagated from the penultimate slice with the complex value of the of final slice, and so define our 'exit' plane as the z-position of the final slice.

If we then imagine that this wave is then projected onto a TEM viewing screen or camera, we would be looking at an image with an intensity that is proportional the squared amplitude of the complex wave. Common practice in TEM imaging is to take an FT of this intensity image to learn about the frequency content of the image. Thus, to give an image similar to those produced in TEM imaging, we also take the square of the amplitude (which gives the intensity) and take an FT from this to learn about the frequency content of our images and reconstructions. This allows more consistent comparison with conventional TEM imaging, and is used to form the FTs presented in Fig. 1(c), which for convenience we reproduce in Supplementary Fig. 6 (a). It is also common to apply some gamma adjustment to FTs, and here we apply a gamma correction of 0.9 to the amplitude presented in Figures 1(c), 3(b), and Supplementary Fig. 6 (a).

An alternative approach to presenting the results of multi-slice ptychography is to present the summed phase of the individual slices in the reconstruction[19]. However, in crystalline samples composed of heavier elements such as the gold used in our experiments, there is also significant contrast in the produced amplitude image. Thus, for such samples it might be more appropriate to look at the amplitude image alongside or perhaps in preference to the phase image when seeking insights into the specimen structure, which we investigate here.

There are a variety of ways in which multiple complex valued slices can be combined for presentation into a single real valued image, and also multiple ways that a complex valued FT can be presented as a single image (with real values). Here for comparison, we produce FTs in a variety of ways, presenting them in Supplementary Fig. 6(x), where sub-figure (x) listed below, is the amplitude of the FT of the:

(a) exit-wave intensity (square of the amplitude);
(b) exit-wave phase, where the phase is represented as a real number before taking its FT;
(c) complex exit-wave;
(d) square of the amplitude of the product of the amplitude of the individual slices;
(e) sum of the phases of the individual slices;
(f) complex product of the individual complex slices.

When we compare the FTs of the simple sums or products (d, e, f), to those of the exit-waves (a, b, c) we see that the relative ring intensities of the exit-wave FTs (a, b, c) follow the diffraction ring intensity trend expected from TEM image simulation and diffraction ring intensity modelling. For example, looking at Supplementary Fig. 2(a), the plotted radial average (blue line) is taken from the FT of (a) above, and the relative ring intensities follow that expected from modelling randomly oriented gold crystals (orange line). However, the FTs of the summed phase shifts, products of amplitudes, or complex products (d – f), show relative ring intensities that do not follow the expected trends from the modelling. Thus, if one were to naively look at these FTs (d – f), one would come to the incorrect conclusion that the crystallites contained in the gold particles had some preferred orientation direction, or other structural anomaly. For example, notice the diminished visibility of the {113}

rings in Supplementary Fig. 6 (d – f) compared to those in (a – c) (the {113} ring is the 4th from centre at 0.813 $Å^{-1}$). Thus, as conventional TEM observations of our gold particles show that they have random orientations and no structural anomalies, the presentations (a – c) using calculated exit-waves (rather than simple sums slice phases as for example used elsewhere[19]) appear as the most directly interpretable FT representations of the reconstructions for our purposes.

We also propose that our use of a propagated wave as opposed to summed phase gives the best representation of the sample and the expected trend in diffraction peak intensities in the FT (see Supplementary Fig. 6a, and Fig.2 blue line and dashed black line), due to us performing our multi-slice reconstruction with a slice spacing (Equation S.4) that is towards the allowable upper limit for a valid reconstruction[51]. In this case the individual slices of the reconstruction have a less directly interpretable meaning than is the case when the slice separation is significantly below this limit. When the slice separation is well below this limit, as has been used in most current high-resolution ptychographic studies of crystalline samples, the summed phase of the slices appears to match well with that expected of the system. When operating with a slice thickness towards the upper limit, one may consider the individual slices as a means to produce accurately matched diffraction data, but the depth resolved information they convey is not so clear. A little more precisely, one could perhaps imagine that the coarsely separated slices of our model contain a sum of Fresnel propagated waves scattered from the atomic structure potentials that would be more clearly evident in imaginary finer separated slices that represent the sample placed between our coarsely separated slices.

This may help explain why propagating a plane wave through our slices gives an exit wave with the expected trend in diffraction plane intensities, whereas the summed phase does not match as well with model expectations (produced from CrystalMaker software as described in Methods). There does not appear to have been any problem with unwrapping the phases of the individual slices before we performed the phase summing to compute our phase summed images and their FTs. Such problems, if present, might have been a source of discrepancy between our models and experimental reconstructions. To aid further comparison we have also presented images of the summed phase of regions of our sample in Supplementary Fig. 11. As would be expected from the FTs presented in Supplementary Fig. 6, the visibility of some high-resolution information is diminished in the summed phase presentation.

4. Simulations

Two types of multislice simulations were performed for this paper: 4D-scanning transmission electron microscopy (4D-STEM) simulations involving multilayer MoS_2 systems, and high-resolution transmission electron microscopy (HRTEM) simulations involving Au/MoS_2 systems. All simulations were performed using

PyPrismatic 2.0 software[76], which uses the PRISM algorithm[77,78]. Our simulations using this software were aided by our own Python wrapper code, Prismatique[79].

4.1 Estimating Layer Count and Tilt of Experimental MoS_2 via Template Matching

To estimate the layer count and tilt of the experimental MoS_2 sample, we generated spatially averaged simulated convergent beam electron diffraction (CBED) patterns for a variety of model MoS_2 systems, and for each spatially averaged simulated CBED pattern we compared a fixed subset of relative integrated CBED disk intensities to those of a spatially averaged experimental CBED pattern. The correlation between the experimentally obtained subset of relative integrated CBED disk intensities and those from a given simulation was quantified using the Pearson correlation coefficient (PCC), with a larger PCC indicating a better match.

We considered model MoS_2 systems containing 1 to 6 layers, polar angles from 0° to 3.6° at steps of 0.05°, and azimuthal angles from 0° to 360° at steps of 0.5°. We assumed a 2H stacking of the MoS_2 layers, with two layers per unit cell, and all layers in the system being equally spaced. The top view of a model bilayer MoS_2 system with no tilt applied is depicted in e.g. Figure 3(a) of Gan, *et al*[80]. We chose an orthorhombic unit cell with one unit cell lattice vector of length a pointing along the x-axis, and another of length $3^{1/2}a$ pointing along the y-axis, where a is the nearest neighbour spacing between intralayer Mo atoms, which we assumed to be equal to 3.1604 Å. The remaining unit cell lattice vector points along the z-axis, and is of length c = 12.295 Å.

We found best correlation with our experimental data when 5 layers were used in the model. Comparing ratios of the integrated intensities of diffraction discs relates to low normalized spatial frequencies (<0.1) in the modulation transfer function (MTF) of the detector, owing to the relatively large size (~20 pixels) of the diffraction discs. Though we did not characterize the MTF of our pixelated direct detector at 20 keV, we suspect and assumed it would be similar to the near ideal characteristics displayed by Medipix detector at 60 keV [81], owing to detectors having similar physical arrangements, albeit with our detector having a larger (75 µm versus 55 µm) pixel size[62]. Owing to the expected flatness in this low frequency-end of the MTF, we did not consider it necessary to apply any Stobb's-like correction factor to in order match our simulations and experiments[82].

Each model system was constructed as follows: First, the orthorhombic unit cell was tiled 57 times in the x-direction, 33 times in the y-direction, and $N_L/2$ times in the z-direction, where N_L is the total number of layers in the system; Secondly, the system was tilted about the geometric center of the resulting collection of atoms; Thirdly, we defined an orthorhombic supercell with edges parallel to the Cartesian axes and dimensions chosen such that the distance between each supercell face and the atom nearest to it was 3 Å; Lastly, periodic boundary conditions were applied to the supercell.

In Table S.1, we list the simulation parameters that are fixed for all multislice simulations, including the high-resolution HRTEM simulations involving Au/MoS_2 systems. Most of the parameters listed should be self-explanatory except for the following: The atomic potential extent refers to the radial distance from the center of any atom, beyond which the potential of said atom is set to zero; and the z-supersampling refers to the number of equally spaced quadrature points to use in performing the explicit 3D integrations of the atomic potentials to obtain the potential slices of the model system. The remaining parameters of relevance used in the 4D-STEM simulations involving multilayer MoS_2 systems are listed in Table S.2.

4.2 HRTEM Simulations Involving Au/MoS_2 Systems

For the HRTEM simulations, we considered one system consisting of a Au island on multilayer MoS_2 with no tilt applied, and another system identical to the former except for an applied tilt. The Au/MoS_2 system with no tilt applied was constructed as follows: First, the orthorhombic unit cell of MoS_2 described in the previous section was tiled 71 times in the x-direction, 41 times in the y-direction, and 2.5 times in the z-direction, yielding 5 layers of MoS_2; Secondly, we constructed a Au island subsystem which had a hexagonal frustum geometry, where the circumradii of the bottom and top faces were approximately 5 nm and 3.75 nm respectively, the height was approximately 3.2 nm, and the (111) planes of the Au subsystem were parallel to the bottom and top faces; Thirdly, the Au island was positioned and oriented such that the bottom face of the island was parallel to layers of MoS_2, the distance between bottom face of the island and the nearest MoS_2 layer was 3 Å, the Au atom closest to the lateral center of the bottom face of the island shared the same lateral coordinates as those of the S atom closest to the lateral center of the MoS_2 subsystem, one of the edges of the bottom face of the island ran parallel to the x-axis, and the Au atoms defining said edge were spaced apart by the next nearest neighbour distance of Au. Fourthly, we defined an orthorhombic supercell with edges parallel to the Cartesian axes and dimensions chosen such that the distance between each supercell face and the atom nearest to it was 3 Å; Lastly, periodic boundary conditions were applied to the supercell.

The tilted Au/MoS_2 system was constructed as follows: First we followed steps 1, 2, and 3 described in the previous paragraph; Secondly, the Au/MoS_2 system was tilted about the geometric center of the MoS_2 subsystem with polar and azimuthal angles 2.95° and 84° respectively; Thirdly, we defined an orthorhombic supercell with edges parallel to the Cartesian axes and dimensions chosen such that the distance between each supercell face and the atom nearest to it was 3 Å; Lastly, periodic boundary conditions were applied to the supercell.

Tables S.1 and S.3 list the parameters of relevance used in all HRTEM simulations

.

Parameter	Value
atomic potential extent	3 Å
explicit 3D potential integration enabled	True
z-supersampling	16
beam energy	20 keV
RMS x-displacement of Mo atoms	0.069 Å
RMS x-displacement of S atoms	0.062 Å

Table S.1 – Fixed simulation parameters for all multislice simulations.

Parameter	Value
lateral dimensions of potential slices	1024x1024 pixels^2
thermal effects enabled	True
# of frozen phonon configurations	1
target slice thickness	1 Å
convergence angle (α)	4.84 mrads
C_s	8 mm
defocus	$-(C_s/10^{-7})(\alpha/10^3)^2-(X/6)(\alpha/10^3)$
scan step size along x-direction	1 Å
scan step size along y-direction	1 Å
# probe positions along x-direction	5
# probe positions along y-direction	5

Table S.2 – Parameters used in 4D-STEM simulations involving multilayer MoS_2 systems not listed in Table S.1. X is the x-dimension of the multilayer MoS_2 supercell in units of Å. In the convention that we adopt in this paper, a negative defocus corresponds to an underfocused microscope.

Parameter	Value
lateral dimensions of potential slices	4096x4096 $pixels^2$
thermal effects enabled	False
target slice thickness	0.9 Å
C_s	8 mm
defocus	0 Å

Table S.3 – Parameters used in HRTEM simulations involving Au/MoS_2 systems not listed in Table S.1.

4.3 Single Slice Test Object and Simulations

The single slice synthetic test object (phase shown in Supplementary Fig. 8 a, b) was prepared using Adobe Illustrator software to produce a bitmap with a wide range of spatial frequencies which was read into Matlab to produce and overlay a series of double-gaussian peaks with variable separations at well separated locations throughout the image. Example double-peak features are at x = 24 nm, y = 26 nm in Supplementary Fig. 8 a, b, c, d and shown with small bounding rectangle, which is shown in the expanded views of Supplementary Fig. 8 h, i, and j. The object was computationally 'illuminated' with a beam of the same energy (wavelength) as in our experiments, and with convergence half-angles, α, defoci, and overlap that were approximately in the same range as our experimental conditions. Specifically, α = 7.5, 10, 15 and 20 mrad were used in the simulations, where the latter two of these conditions would have resulted in overlap between the Bragg diffracted beams from their respective samples (see Supplementary Fig. 8). These data were then reconstructed using the same parameters as for our experimental reconstruction of the Au-aC or Au-MoS_2 sample. The resulting reconstructed phase was then Fourier transformed and compared to the phase of our source object to determine a radially averaged signal to noise ratio (SNR), using the method in Equation 44 of Thibault *et al.*[83], and phase contrast transfer function (PCTF) (Supplementary Fig. 8 e, f).

The PCTF and SNR calculations are implemented in TestScripts \ Part_5_PCTF_SNR_on_SyntheticData.m of our related code repository (https://github.com/ArthurBlackburn/PtychoRunner)[68].

5. Fourier Ring Correlation Determination

The FRC was determined by splitting the ptychographic dataset into two subsets, forming the subsets from the even and odd numbered diffraction patterns (DP) as acquired, respectively. Reconstructions were produced for each subset, using the same recipe as the full dataset reconstruction. These two reconstructions were effectively formed at half the electron dose of the full set. This method of producing independent subsets reduces the influence of sample drift and electromagnetic-noise induced beam-position movement upon on the FRC, as discussed and used in prior work[19,84]. The Fourier Ring Correlation (FRC) metric produces a resolution metric from two independent images or reconstructions of a sample, as discussed by van Heel and Shatz[46]. The FRC is defined as

$$FRC(r_i) = \frac{\sum_{r \in r_i} F_1(r) \cdot F_2(r)^*}{\sqrt{\sum_{r \in r_i} F_1^2(r) \cdot \sum_{r \in r_i} F_2^2(r)}}. \tag{S.7}$$

Here r is the spatial frequency, $F(r)$ is the complex value at position r in the Fourier transform of the image, and * denotes complex conjugation. The summations are over all Fourier-space pixels r that are contained in the shell r_i. When this is applied to images comprised of isolated particles on an unstructured support material such as amorphous ice or carbon, a form of masking around the particles is commonly applied in the analysis of the images so that the expected lack of correlation in the unstructured background does not unduly cause underestimation of the resolution[50]. Alternatively, it is also acceptable to analyze sub-regions of an image, by sweeping a window through the image[85], or use a modified definition of the FRC where a localized resolution measure can be gained[59].

Here we use a swept window approach, comparing 512 × 512 pixel sub-regions of the independent reconstructions, extracted from the full reconstruction on a regular 8 × 8 grid. This produces 64, 512 × 512 pixel sub-regions. The whole images were aligned and the FRC characteristic was determined using code provided within our github repository[68] and the PtychoShelves package[64], and as described by Vila-Comamala[45]. In the sub-regions which were dominated by gold particles, the mean FRC over all spatial frequencies, is greater than in the regions which were dominated by the amorphous carbon support. A histogram of the mean FRC in the 64 sub-regions is given in Supplementary Fig. 7.

This distribution appears to indicate two groups: regions dominated by the presence of gold particles, and regions dominated by the amorphous background support. To eliminate the contribution to our resolution measure of subregions that had little or no useful information (such as regions containing mainly amorphous

carbon), regions with a mean FRC below a threshold given by Otsu's method were excluded from further consideration. Otsu's method gives a threshold that minimizes the intra-class variance in the distribution and is a standard and well-known method of thresholding on such distributions[86]. Here Otsu's method gave the threshold mean FRC score as 0.17, which is indicated in Supplementary Fig. 7(b) with a red dashed line. Thus 18/64 (28%) of the regions were excluded, but we noted that this was considerably less than the fraction of the image that was comprised solely of amorphous carbon. Our method reduced the dependence of the resolution measure on the incomplete gold-particle coverage in the field of view, and produced a more reliable, repeatable and meaningful resolution measure. This effect and principle is similar to masking[50], swept window approaches[85], and localized FRC resolution measure determination[59], which are used in practical applications of FRC.

If we did not adopt some means of thresholding and making adjustment for the fact our sample contained a sparse distribution of particles, then we would have a resolution measure that depended to some extent on the coverage of particles in the field of view. Our sub-region selection strategy, thus has a similar effect as masks used to calculate the FRC for cryo-EM imaging resolution determination[59]. The effect is to reduce the dependence of the resolution measure on the sample coverage in the field of view, and to thus produce a more reliable, repeatable and meaningful resolution measure.

The mean of the FRC characteristics from the subregions above the Otsu threshold value is presented in the main text (Fig. 2). In Supplementary Fig. 7(a) the centres of the sub-regions that are included in the presented mean FRC characteristic are indicated with an ○ and those that are excluded with an ×. These markers are overlaid on a color-scale image of the reconstructed phase of one of the independent reconstructions. The reconstruction used half of the diffraction pattern set used to produce the final reconstruction given in Fig. 1 of the main text, but followed the same recipe as given in Table 1. It is evident from Supplementary Fig. 7(a) that the excluded regions fall in regions primarily of amorphous carbon. An illustration of the bounds of a typical excluded sub-region is also shown in this figure as a black square.

The resolution measure is extracted from the FRC by determining the highest spatial frequency at which it intersects a noise threshold level. Here, we used the ½ bit threshold which corresponds to a flat signal to noise ratio of 0.41 in the Fourier domain[45]. This threshold has been applied to other ptychographic resolution measures[17,18,45,47], and in general is appropriate when the final image used is the average or sum of the two individual images. Summing two images effectively doubles the exposure dose, and similarly the full reconstruction presented here (Fig. 1) has double the exposure dose of the compared independent reconstructions. Our full reconstruction also has twice the sampling density of the independent reconstructions, and it has been observed that the Fourier error decreases with increased sampling density or overlap parameter[87].

An increased sampling density (which can be varied independently of dose) giving a reduced Fourier error, might lead to a further increase in resolution beyond that attributed to the increasing exposure dose[21], and thus perhaps justify some decrease in the noise threshold level below the ½ bit level. We have not verified this, but if true it would indicate our resolution measure of 67 pm is conservative, as is also suggested by resolving 63 pm feature separations in the Au/MoS_2 reconstruction.

References

References 15 – 68 below are also as in the main text but are repeated here for convenience and in case any variations in order appear during the main text production process.

15 Blackburn, A. M. & McLeod, R. A. Practical implementation of high-resolution electron ptychography and comparison with off-axis electron holography. *Microscopy* **70**, 131-147 (2020). https://doi.org/10.1093/jmicro/dfaa055

17 Allars, F. *et al.* Efficient large field of view electron phase imaging using near-field electron ptychography with a diffuser. *Ultramicroscopy* **231**, 113257 (2021). https://doi.org/10.1016/j.ultramic.2021.113257

18 Pelz, P. M., Qiu, W. X., Bücker, R., Kassier, G. & Miller, R. J. D. Low-dose cryo electron ptychography via non-convex Bayesian optimization. *Scientific Reports* **7**, 9883 (2017). https://doi.org/10.1038/s41598-017-07488-y

19 Chen, Z. *et al.* Electron ptychography achieves atomic-resolution limits set by lattice vibrations. *Science* **372**, 826 (2021). https://doi.org/10.1126/science.abg2533

21 Jiang, Y. *et al.* Electron ptychography of 2D materials to deep sub-ångström resolution. *Nature* **559**, 343-349 (2018). https://doi.org/10.1038/s41586-018-0298-5

26 Maiden, A. M. & Rodenburg, J. M. An improved ptychographical phase retrieval algorithm for diffractive imaging. *Ultramicroscopy* **109**, 1256-1262 (2009). https://doi.org/10.1016/j.ultramic.2009.05.012

45 Vila-Comamala, J. *et al.* Characterization of high-resolution diffractive X-ray optics by ptychographic coherent diffractive imaging. *Optics Express* **19**, 21333-21344 (2011). https://doi.org/10.1364/OE.19.021333

46 van Heel, M. & Schatz, M. Fourier shell correlation threshold criteria. *Journal of Structural Biology* **151**, 250-262 (2005). https://doi.org/10.1016/j.jsb.2005.05.009

47 Jiang, Y. *et al.* Achieving high spatial resolution in a large field-of-view using lensless x-ray imaging. *Applied Physics Letters* **119** (2021). https://doi.org/10.1063/5.0067197

50 Penczek, P. Reliable cryo-EM resolution estimation with modified Fourier shell correlation. *IUCrJ* **7**, 995-1008 (2020). https://doi.org/10.1107/S2052252520011574

51 Tsai, E. H. R., Usov, I., Diaz, A., Menzel, A. & Guizar-Sicairos, M. X-ray ptychography with extended depth of field. *Optics Express* **24**, 29089-29108 (2016). https://doi.org/10.1364/OE.24.029089

53 Thibault, P. & Menzel, A. Reconstructing state mixtures from diffraction measurements. *Nature* **494**, 68 (2013). https://doi.org/10.1038/nature11806

59 Kucukelbir, A., Sigworth, F. J. & Tagare, H. D. Quantifying the local resolution of cryo-EM density maps. *Nature Methods* **11**, 63-65 (2014). https://doi.org/10.1038/nmeth.2727

62 Tinti, G. *et al.* The EIGER detector for low-energy electron microscopy and photoemission electron microscopy. *Journal of Synchrotron Radiation* **24**, 963-974 (2017). https://doi.org/10.1107/S1600577517009109

63 Odstrčil, M., Menzel, A. & Guizar-Sicairos, M. Iterative least-squares solver for generalized maximum-likelihood ptychography. *Optics Express* **26**, 3108-3123 (2018). https://doi.org/10.1364/OE.26.003108

64 Wakonig, K. *et al.* PtychoShelves, a versatile high-level framework for high-performance analysis of ptychographic dataThis article will form part of a virtual special issue of the journal on ptychography software and technical developments. *Journal of Applied Crystallography* **53**, 574-586 (2020). https://doi.org/10.1107/S1600576720001776

66 Jezzard, P. & Balaban, R. S. Correction for geometric distortion in echo planar images from B0 field variations. *Magnetic Resonance in Medicine* **34**, 65-73 (1995). https://doi.org/10.1002/mrm.1910340111

68 Blackburn, A. M. Codes for realization of sub-ångström resolution ptychography in a scanning electron microscope at 20 keV. Zenodo (2025). https://doi.org/10.5281/zenodo.16957173

69 Rodenburg, J. M. & Bates, R. H. T. The Theory of Super-Resolution Electron Microscopy Via Wigner-Distribution Deconvolution. *Philosophical Transactions: Physical Sciences and Engineering* **339**, 521-553 (1992). https://doi.org/10.1098/rsta.1992.0050

70 Holler, M. *et al.* X-ray ptychographic computed tomography at 16 nm isotropic 3D resolution. *Scientific Reports* **4**, 3857 (2014). https://doi.org/10.1038/srep03857

71 Li, P. & Maiden, A. Multi-slice ptychographic tomography. *Scientific Reports* **8**, 2049 (2018). https://doi.org/10.1038/s41598-018-20530-x

72 Goswami, A. & Lisgarten, N. D. The measurement of inner potentials for copper, silver and gold. *Journal of Physics C: Solid State Physics* **15**, 4217 (1982). https://doi.org/10.1088/0022-3719/15/19/020

73 Dwivedi, P., Konijnenberg, A. P., Pereira, S. F. & Urbach, H. P. Lateral position correction in ptychography using the gradient of intensity patterns. *Ultramicroscopy* **192**, 29-36 (2018). https://doi.org/10.1016/j.ultramic.2018.04.004

74 Maiden, A. M., Humphry, M. J., Sarahan, M. C., Kraus, B. & Rodenburg, J. M. An annealing algorithm to correct positioning errors in ptychography. *Ultramicroscopy* **120**, 64-72 (2012). https://doi.org/10.1016/j.ultramic.2012.06.001

75 Tyson, R. K. Conversion of Zernike aberration coefficients to Seidel and higher-order power-series aberration coefficients. *Optics Letters* **7**, 262-264 (1982). https://doi.org/10.1364/OL.7.000262

76 Rangel DaCosta, L. *et al.* Prismatic 2.0 – Simulation software for scanning and high resolution transmission electron microscopy (STEM and HRTEM). *Micron* **151**, 103141 (2021). https://doi.org/10.1016/j.micron.2021.103141

77 Pryor, A., Ophus, C. & Miao, J. A streaming multi-GPU implementation of image simulation algorithms for scanning transmission electron microscopy. *Advanced Structural and Chemical Imaging* **3**, 15 (2017). https://doi.org/10.1186/s40679-017-0048-z

78 Ophus, C. A fast image simulation algorithm for scanning transmission electron microscopy. *Advanced Structural and Chemical Imaging* **3**, 13 (2017). https://doi.org/10.1186/s40679-017-0046-1

79 Fitzpatrick, M. R. C. Prismatique (2023).https://gitlab.com/mrfitzpa/prismatique

80 Gan, X. *et al.* 2H/1T Phase Transition of Multilayer MoS2 by Electrochemical Incorporation of S Vacancies. *ACS Applied Energy Materials* **1**, 4754-4765 (2018). https://doi.org/10.1021/acsaem.8b00875

81 Mir, J. A. *et al.* Characterisation of the Medipix3 detector for 60 and 80keV electrons. *Ultramicroscopy* **182**, 44-53 (2017). https://doi.org/https://doi.org/10.1016/j.ultramic.2017.06.010

82 Thust, A. High-Resolution Transmission Electron Microscopy on an Absolute Contrast Scale. *Physical Review Letters* **102**, 220801 (2009). https://doi.org/10.1103/PhysRevLett.102.220801

83 Thibault, P. & Guizar-Sicairos, M. Maximum-likelihood refinement for coherent diffractive imaging. *New Journal of Physics* **14**, 063004 (2012). https://doi.org/10.1088/1367-2630/14/6/063004

84 Ding, Z. *et al.* Three-dimensional electron ptychography of organic–inorganic hybrid nanostructures. *Nature Communications* **13**, 4787 (2022). https://doi.org/10.1038/s41467-022-32548-x

85 Hashem, Y. *et al.* High-resolution cryo-electron microscopy structure of the Trypanosoma brucei ribosome. *Nature* **494**, 385-389 (2013). https://doi.org/10.1038/nature11872

86 Otsu, N. A Threshold Selection Method from Gray-Level Histograms. *IEEE Transactions on Systems, Man, and Cybernetics* **9**, 62-66 (1979). https://doi.org/10.1109/TSMC.1979.4310076

87 Bunk, O. *et al.* Influence of the overlap parameter on the convergence of the ptychographical iterative engine. *Ultramicroscopy* **108**, 481-487 (2008). https://doi.org/10.1016/j.ultramic.2007.08.003
